# Kinetic Assessment and Therapeutic Modulation of Metabolic and Inflammatory Profiles in Mice on a High-Fat and Cholesterol Diet

**DOI:** 10.1155/2010/970164

**Published:** 2010-04-22

**Authors:** Laura W. Engstrom, Loretta Bober, Shu-Cheng Chen, Jay S. Fine, Ying Li, Michaela C. Stanton, David Kinsley, Long Cui, James V. Jackson, Alberto Rojas-Triana, Daniel Lundell, Maureen Laverty, Eric L. Gustafson, Chung-Her Jenh, Timothy J. Kowalski, Denise J. Manfra

**Affiliations:** ^1^Department of Inflammation, Schering-Plough Research Institute, Kenilworth, NJ 07033, USA; ^2^Department of Cardiovascular and Metabolic Disease, Schering-Plough Research Institute, Kenilworth, NJ 07033, USA; ^3^Department of In vivo and Translational Biology, Hoffman-LaRoche, Nutley, NJ 070110, USA; ^4^Department of Respiratory, Inflammation, and Autoimmunity, MedImmune, Gaithersburg, MD 20878, USA; ^5^Discovery Technologies Department, Schering-Plough Research Institute, Kenilworth, NJ 07033, USA

## Abstract

The kinetics of metabolic and inflammatory parameters associated with obesity were evaluated in a murine diet-induced obesity (DIO) model using a diet high in fat and cholesterol. Cellular infiltration and mediator production were assessed and shown to be therapeutically modulated by the PPARgamma agonist rosiglitazone. C57BL/6 mice were maintained on a 45% fat/ 0.12% cholesterol (HF/CH) or Chow diet for 3, 6, 16, or 27 weeks. Flow cytometry was employed to monitor peripheral blood monocytes and adipose tissue macrophages (ATM). Gene expression and protein analysis methods were used to evaluate mediator production from total epididymal fat (EF), stromal vascular fraction (SVF), and sorted SVF cells. To investigate therapeutic intervention, mice were fed a HF/CH diet for 12 weeks and then a diet formulated with rosiglitazone (5 mg/kg) for an additional 6 weeks. A HF/CH diet correlated with obesity and a dramatic proinflammatory state. Therapeutic intervention with rosiglitazone attenuated the HF/CH induced inflammation. In addition, a novel population was found that expressed the highest levels of the pro-inflammatory mediators CCL2 and IL-6.

## 1. Introduction

In a Western society, where diets are generally high in fat and cholesterol, 33% of adults and 17% of children have been classified as obese [1, 2]. Obesity and its associated disorders, including insulin resistance, type II diabetes, nonalcoholic fatty liver disease, and atherosclerosis, are growing medical concerns [3]. Chronic low-grade inflammation is proposed as an important link between obesity and its associated pathologies [1, 3, 4]. Data from both humans [5, 6] and mice [6–9] have shown a positive correlation between increasing BMI body mass index (BMI) and hyperinsulinemia, and macrophage content in the adipose tissue. 

In both human [6, 10, 11] and mouse [6, 8, 9, 12–14], various studies have shown an inflammatory reaction in adipose tissue characterized by macrophage accumulation and increased levels of mediators including TNF*α*, IL-1*β*, IL-6, IL-10, and MCP-1. Macrophages are proposed to be a major source of pro-inflammatory mediators, and the current dogma suggests the existence of two subsets of murine adipose tissue macrophages (ATM). Pro-inflammatory macrophages (PI-ATM) express TNF*α*, IL-1*β*, IL-4, IL-6, CCL2, and CCR2, and the alternatively activated/resident macrophages (R-ATM) express IL-10, ApoE, and arginase [6, 8, 9, 12–14]. In contrast, human ATMs have been reported to have an alternatively activated surface phenotype, yet secrete both pro- and anti-inflammatory mediators. [11, 15, 16]. 

Currently, most of the murine models of obesity used to study the inflammatory component of obesity have employed diets rich in fat only. However, humans often consume diets that are rich in cholesterol as well as fat that are associated not only with obesity but also with two comorbidities of obesity such as nonalcoholic fatty liver disease (NAFLD) and atherosclerosis both of which are also associated with underlying chronic low grade inflammation [3, 4, 17–19]. NAFLD is also associated with insulin resistance [17]. It has been reported previously that feeding mice a high-fat and cholesterol (HF/CH) diet has been shown to induce obesity as well as elevate systemic levels of alanine aminotransferase (ALT) and cause liver damage, indicative of nonalcoholic fatty liver disease (NAFLD) [2]. Our goal was to define the impact of a high-fat and cholesterol diet in the DIO model on inflammatory and metabolic parameters by profiling mediator production and by monitoring the modulation of peripheral and adipose tissue immune cells. Although it has been shown in both rodents and humans that diet and/or exercise can modulate certain inflammatory parameters [20–23], in some cases pharmacologic intervention may still be necessary. Therefore, the second aim was to determine the utility of the model for discovery of type II diabetes and metabolic syndrome drugs by evaluating a known clinical glycemic modulating drug, rosiglitazone. Recent studies have shown that in addition to modulating glycemic levels, rosiglitazone also has immunomodulatory activity in adipose tissue [8, 24–27]. 

 The manuscript describes the kinetics of multiple inflammatory parameters associated with the development of obesity. Specifically, we assessed the kinetics of blood monocytes and ATMs from DIO mice, and the modulation of these leukocytes by rosiglitazone. Furthermore, we report on the identification of two novel adipose tissue cellular infiltrates capable of producing inflammatory mediators.

## 2. Materials and Methods

### 2.1. Model Establishment

C57Bl/6 mice (Charles River) at 6-weeks of age were placed either on a high-fat diet consisting of 45% fat and 0.12% cholesterol (D0401280, Research Diets Inc, New Brunswick, NJ) or a Chow diet (PicoLab 5053, Lab Diet) (Table 1). The high-fat diet from Research Diets Inc. is built on their basic diet D12451 with the protein content at 20% kcal, the carbohydrates 35% kcal, and the fat 45% kcal. The fatty acid profile of this diet is 36.3% saturated, 45.3% monosaturated, and 18.5% polyunsaturated fat. The diet intake per mouse per day is ~3 grams or 14.16 kcal per day (4.72 kcal/gm ∗ 3 grams). Animals were on a 12 : 12 light dark cycle and given food ad libitum. Experiments were conducted under our institutional guidelines for the humane treatment of laboratory animals. 

 Metabolic parameters and flow cytometric analysis were assessed on two separate cohorts of animals from the same study. All animals were analyzed for changes in body weight (BW), epididymal fat pad mass, and total fat and lean mass. Total fat and lean mass were determined 1 week prior to each termination time point using the EchoMRI-100 for mice (ECHOMRI, Echo Medical Systems, LLC Houston TX). 

 Rosiglitazone was formulated at 5 mg/kg into the diet calculated on the basis of predetermined food intake per mouse (~3 grams). The dose of 5 mg/kg was chosen based on in-house data demonstrating significant improvement in insulin sensitivity as assessed by insulin tolerance test (ITT). Animals were fed an HF/CH diet for 12 weeks before being switched to the HF/CH diet with drug for an additional 6-weeks. Food was available ad libitum for the mice. Insulin and leptin levels were measured prior to initiation of drug dosing and then again at termination. 

### 2.2. Metabolic Parameters

Serum samples were collected into T-MG Capiject tubes (Terumo Medical Corp, Elkton, MD) via cardiac puncture. Serum samples were analyzed using MesoScale Diagnostics assays (Meso Scale Discovery, Gaithersburg, MD): pro-inflammatory mediator kit (Cat # K11012b-2), adiponectin kit (Cat # K112BXC-2), and a custom metabolic multiplex (IL-6, GM-CSF, insulin, leptin, resisten, and TNF*α*). Insulin and leptin were nonfasting levels measured at termination. ALT was assessed by an ALT enzymatic assay (Catachem # C164-02 Bridgeport, CT). 

 The epididymal fat pads were collected in KHB buffer (Krebs-bicarbonate-HEPES, pH7.4) (Celsis In Vitro Technologies, Chicago, IL) containing 4% essentially fatty acid-free bovine serum albumin (Sigma-Aldrich, St. Louis, MO) 5 mM glucose (Sigma-Aldrich), and 1 X Penicillin-Streptomycin (Invitrogen Corporation, Carlsbad, CA). The tissue was digested with the described buffer using 1 mg/ml collagenase type 1 (Worthington Biochemical Corp. Lakewood, NJ) with 0.1 mg/ml DNase-1 (Sigma-Aldrich) and then passed through a 250 *μ*m nylon mesh (Small Parts, Inc., Miramar, FL) and a 100 *μ*m cell strainer (BD Biosciences, Bedford MA). After centrifugation, the pellet containing the SVF was resuspended in RPMI 1640 (Invitrogen) containing 10% FBS (Gemini Bio-Products, West Sacramento, CA), and penicillin/streptomycin (Invitrogen), and washed twice. 1 × 10^5^ cells were added per well in a 48 well tissue culture plate and incubated at 37°C, 5% CO_2_ for 24 hours. Culture supernatants were analyzed using MesoScale Diagnostics multiplex kits (Meso Scale Discovery, Gaithersburg, MD). Statistical analysis was determined by use of the Mann-Whitney *U* test.

### 2.3. Analysis of Blood and SVF Subpopulations by Flow Cytometry

Epididymal fat was excised and weighed. The SVF was isolated from the epididymal fat using a 45 minute collagenase incubation according to a previously published method [12] and personal communication. Blood was collected from mice into T-MQK Capiject tubes (Terumo Medical Corp) via cardiac puncture. SVF cells or blood leukocytes were blocked with CD16/CD32 (clone 2.4G2, BD Biosciences) and stained with antibodies. For cell sorting, SVF cell suspensions were lysed using RBC Lysis buffer (eBiosciences San Diego, CA) prior to staining; otherwise whole blood and SVF cells were stained and then lysed with BD FACS Lysing solution (BD Pharmingen). Monoclonal antibodies to the following mouse surface antigens were purchased from BD Biosciences CA: Ly6G (1A8), CD11c (HL3), CD3 (145-2C11), B220 (RA-6B2), NK1.1 (PK136), CD45 (30-F11), CD90.2 (53-2.1), CD11b (M1/70), Ly6C (AL-21). Additionally, monoclonal antibodies used were CD62L (clone MEL-14 Invitrogen) and F480 (clone BM8 Ebioscience). Events were acquired on a Becton Dickinson FACsAria and analyzed with FACsDiva Software (BD Biosciences). Sorted cells were collected, pelleted, and stored at −80°C. SVF and blood cells were enumerated with the BD TruCount protocol (Cat # 340498), utilizing the nuclear dye (Control reagent B), and adding antimouse CD45 (BD Biosciences; 30-F11); human antibodies in the kit did not interfere with the detection of mouse CD45 positive leukocytes. Samples were acquired until 4000 beads were collected, and leukocytes were detected by DNA content and CD45. 

### 2.4. RNA Isolation from Epididymal Fat Pad and Quantitative RT-PCR

Epididymal fat tissues were snap-frozen in liquid nitrogen and stored at −80°C. Frozen tissue pieces (<100 mg) were placed in 1 ml of Qiagen QIAzol lysis reagent (Cat # 79306) containing one sterile 5 mm stainless steel ball, and homogenized using two 1-minute cycles at 20 Hz on a TissueLyser (Qiagen, Valencia CA). Total RNA was isolated using Qiagen RNeasy Lipid Tissue mini kit (Cat # 74804) using the additional DNase step according to manufacturer's protocol. RNA was eluted from the column in 60 ul RNase free water and quantified using Nanodrop 1000 v3.3.0 software. cDNA was synthesized using Applied Biosystems High Capacity cDNA reverse transcriptase kit (Cat # 4368813) and quantitated with an 18S standard curve using a TaqMan gene expression assay. Quantitative real-time PCR utilized a custom made TaqMan Low Density Array (TLDA) from Applied Biosystems. 100 ng cDNA was loaded in a 100 ul volume for each port of the TLDA plates. Thermal cycling was performed using an ABI Prism 7900 HT Sequence Detection System and data was analyzed using SDS v2.2 software. The Ct value of each gene is normalized to 18 S to obtain ΔCt. 

### 2.5. RNA Isolation and Quantitative PCR for Sorted Cell Subsets

Total RNA was prepared using the RNAqueous-Micro kit (Ambion, Austin, TX) according to the manufacturer's instructions. TaqMan primers and probes (see Supplementary Table 1 available at doi:10.1155/2010/970164) were designed with Universal Probe Library Assay Design software (Roche, Indianapolis, IN) and purchased from IDT or from Roche, respectively. The PCR reactions were prepared using the components from the iScript Custom One-Step RT-PCR Kit with ROX and assembled according to the manufacturer's instructions (Quanta BioSciences, Gaithersburg, MD). The final concentrations of the primers and probe in the PCR reactions were 200 nM and 100 nM, respectively. The fluorogenic probes were labeled with 6-carboxyfluorescein (6FAM) as the reporter and a nonfluorescent quencher. Each 10 *μ*l PCR reaction contained 2 *μ*l (10 ng) of total RNA. Thermal cycling was performed on an ABI Prism 7900HT Sequence Detection System (Applied Biosystems, Foster City, CA) according to the following protocol: one ten-minute cycle at 50°C, followed by one five-minute cycle at 95°C, followed by forty fifteen-second cycles at 95°C and a final one-minute cycle at 60°C. A eukaryotic 18S rRNA endogenous control probe/primer set (ABI) was used as an internal control for RNA quality. Absolute quantitation of the amount of mRNA in a given sample was determined using a 12-point standard curve generated with 4-fold serial dilutions, starting at 20000 fg, of cDNA containing the gene of interest.

## 3. Results

### 3.1. Kinetic Analysis of the Metabolic Parameters in Mice on an HF/CH Diet

**Figure 1:**
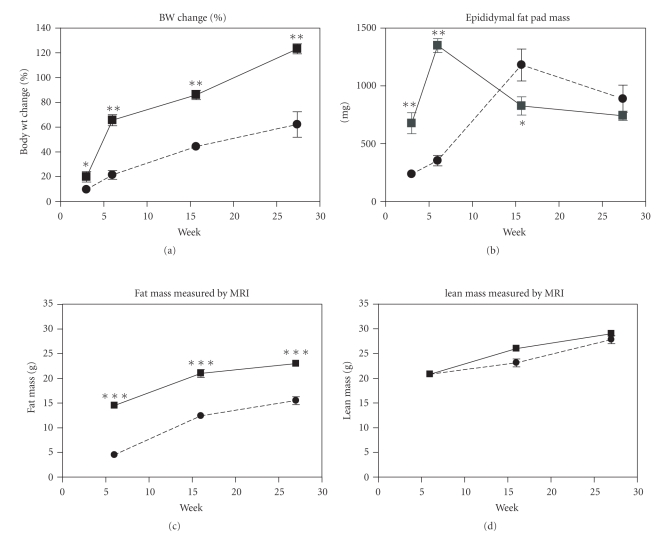
An HF/CH diet kinetically induced metabolic obesity. C57BL/6 mice were placed on Chow (circles and dashed lines) or a diet of 45% fat and 0.12% cholesterol (squares and solid line) for 3, 6, 16, or 27-weeks. Groups of *n* ≥ 7 were sacrificed at each time point for both chow and HF/CH diets. Body weight (a), mg of epididymal fat (b), total fat (c), and lean (d) mass were all measured. Epididymal fat tissue and blood from these animals were subsequently used for FACs analysis. Values represent the mean and SEM. Unpaired *T* test was used to compare Chow to DIO (HF/CH diet cohort) at each time point; *P < .05, **P < .001, and ***P < .0001.

DIO mice became obese after consuming a diet containing 45% fat and 0.12% cholesterol (HF/CH), with the body weight (BW) significantly increased by 3 weeks. By 27-weeks the BW was 120% above their original weight (Figure 1(a)). Similar to body weight, total fat mass (Figure 1(c)) increased steadily. Epididymal fat (EF) mass was significantly elevated early and peaked by 6-weeks (Figure 1(b)) but then declined following a trend similar to other reported data [28] and suggestive of a shift in fat depots. No significant change in lean mass (Figure 1(d)) relative to the Chow cohort was detected. Elevated levels of leptin, insulin, and ALT, an indicator of liver damage, were evident by 6-weeks and remained elevated in the DIO mice compared to the control group at each time point. Serum levels of CCL2 and IL-6 were significantly elevated at 16-weeks, while trending upwards at 6-weeks. Nonsignificant increases in IL-10 were also detected from 6-weeks throughout the study. A HF/CH diet had a minimal effect on serum IL-1*β* and TNF*α* levels (Table 2). 

### 3.2. Progressive Infiltration of Pro-Inflammatory ATMs

**Figure 2:**
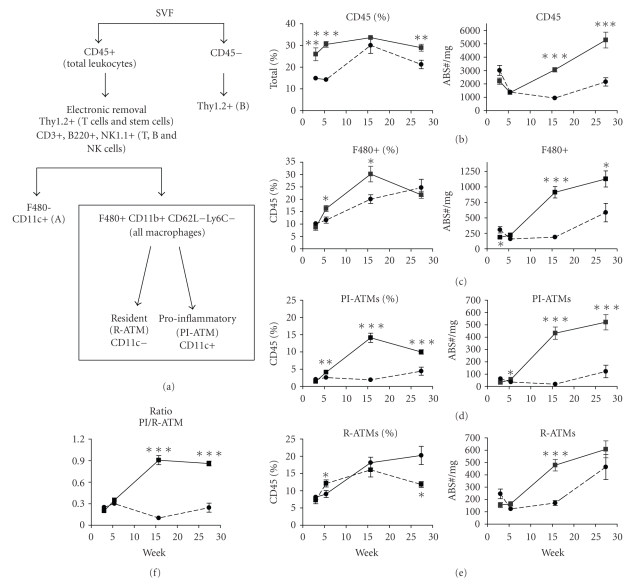
Progressive infiltration of pro-inflammatory ATMs assessed by flow cytometry. The epididymal fat pads were isolated from *n* ≥ 7 animals per group at each time point. The SVF was isolated and stained to identify total leukocytes (b), macrophages (c), the pro-inflammatory (d) and resident (e) ATM subsets according to the gating strategy (a). The ratio of the pro-inflammatory ATMs over the resident ATMs is a quick indicator of the phenotypic profile of the SVF macrophages. Values represent the mean and SEM. The Chow group is represented by circles and dashed lines and the DIO group on HF/CH diet is represented by squares and a solid line. Unpaired *T* test was used to compare standard chow to DIO at each time point; *P ≤ .05, **P ≤ .01, and ***P ≤ .001.

We employed a detailed flow cytometric gating strategy (Figure 2(a)) to evaluate adipose tissue macrophages. Total leukocytes were identified by CD45. In DIO mice, the proportion of these cells increased to ~30% by 3 weeks (Figure 2(b)). Total macrophages were identified as CD45+F480+CD11b+ cells after electronic removal of lymphocytes identified by cell size and the surface antigens CD3, B220, NK1.1, and Thy1.2. The proportion of macrophages was significantly increased in DIO mice by 6-weeks and peaked by 16-weeks (Figure 2(c)). The absolute numbers per mg of fat of both total leukocytes and macrophages progressively increased in the DIO cohort from 16 to 27-weeks. In control mice, there was a modest increase in the proportion of macrophages at 16-weeks and in the absolute numbers per mg of fat of total leukocytes and macrophages at 27-weeks (Figures 2(b) and 2(c)). In contrast to the macrophages, neutrophils were monitored but constituted a small percentage (<7%) of leukocytes and decreased over time (data not shown), consistent with previously published data [29] and a role for neutrophils in the early onset of disease.

Similar increases in macrophage accumulation were observed in the EF pads by immunohistochemistry using anti-F480, and also by gene expression analysis of F480 and CD68 (see Supplementary Figure 1 available at doi:10.1155/2010/970164). Anti-F480 staining of the EF pad from DIO mice showed an accumulation of macrophages and multinucleated crown structures by 6-weeks which were a predominant characteristic of adipose tissue by 16-weeks. A few crown structures were detected in control mice at 27-weeks (see Supplementary Figure 1(a) available at doi:10.1155/2010/970164). Gene expression analysis of F480 and CD68 showed 2–4 fold induction of macrophages by six weeks; macrophage accumulation peaked (7-8 fold) at 16-weeks. At 27-weeks the fold induction was smaller due to the increased number of macrophages in the normal Chow group (see Supplementary Figure 1(b) available at doi:10.1155/2010/970164). 

 To further phenotype the macrophage content in the HF/CH induced DIO model, we profiled the PI-ATM and R-ATM. The total macrophages were delineated as either PI-ATMs (CD11c+) or R-ATM (CD11c-) by the CD11c marker. There was a modest increase in both the PI-ATM and R-ATMs in DIO mice at 6-weeks, with a dramatic increase in the percentage and number of the PI-ATM at 16-weeks (Figures 2(d) and 2(e)). These data correlated with a dramatic increase in the PI/R-ATM ratio, indicating a preferential accumulation of PI-ATM (Figure 2(f)). In contrast, the normal chow mice exhibited a gradual increase in their R-ATM (Figure 2(e)) and a consistently low PI/R-ATM ratio (Figure 2(f)).

### 3.3. Mediator Profile of the Epididymal Tissue, SVF, and Sorted Cells

**Figure 3:**
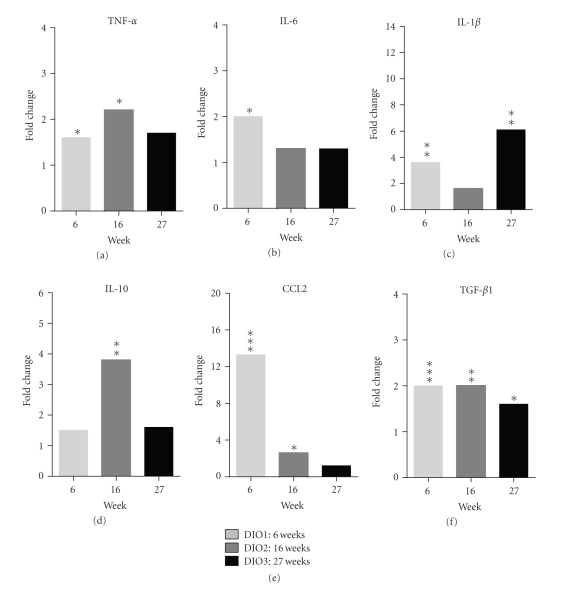
Induction of inflammatory mediators in whole EF assessed by gene expression at 6, 16, and 27-weeks. Data represented as HF/CH gene expression fold induction versus the Chow cohort for each time point. (*n* = 7–10 mice for Chow and *n* = 10–15 mice for the HF/CH group). The light gray bars indicate the 6-week timepoint, the dark gray bars indicate 16-week timepoint, and the black bars indicate the 27-week timepoint. Statistical significance was determined by a two tailed Welch *t*-test; *P < .05, **P < .01, and ***P < .001.

**Figure 4:**
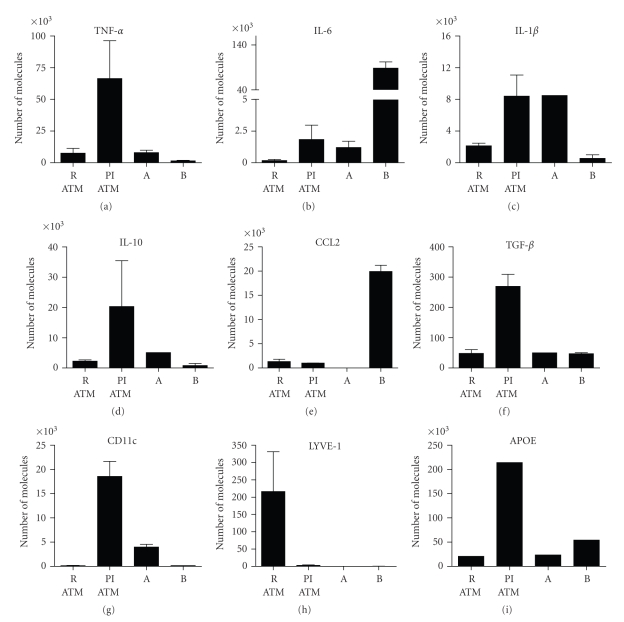
Pro-inflammatory ATMs express both pro- and anti-inflammatory mediators as assessed by gene expression. Four cell populations were sorted from the SVF from mice (*n* ≥ 3) fed an HF/CH diet for approximately 16-weeks. The pro-inflammatory ATMs (PI-ATM: CD45+F480+CD11c+), resident ATMs (R-ATM: CD45+F480+CD11c−), a CD45+ unknown population (A: CD45+F480−CD11c+), and a CD45− unknown population (B: CD45 Thy1.2+Ly6C+/−CD11b−) were sorted and analyzed for gene expression from two independent sorts.

Similar to the mediator profile in serum, elevations in mRNA for CCL2, IL-6, and IL-10 in EF tissue were observed in DIO mice. Detectable increases in TNF*α*, IL-1*β*, and TGF *β* expression in EF were observed as well (Figure 3). Spontaneous mediator production from ex vivo DIO SVF cultures also showed elevated levels of IL-6, IL-10, and TNF*α*, with a minimal increase in IL-1*β* (Figure 6(b)). To determine the contribution of ATMs to mediator production, PI-ATM and R-ATM from DIO mice were sorted based on CD11c expression and profiled by gene expression analysis (Figure 4). In addition to CD11c, PI-ATMs expressed elevated mRNA levels of the characteristic pro-inflammatory mediators, including TNF*α*, IL-6, and IL-1*β*. These cells also expressed elevated levels of ApoE, IL-10, and TGF *β*. LYVE-1 was the only gene expressed at elevated levels in R-ATMs.

### 3.4. Novel Cellular Infiltrates in the Epididymal Fat

Besides macrophages, SVF has been proposed to contain several other cell subsets including preadipocytes, epithelial, and endothelial cells [16, 30]. Our gating strategy led to the detection of two additional populations **(** Figure 2(a)). The first population, identified as CD45+ F480−CD11b+ CD11c+ (A), represented 10–20% of the CD45 population. The second population, identified as CD45−F480−CD11b−Ly6C+/−Thy1.2+ (B), represented 5–10% of total cells of the SVF. Gene expression profiling showed that population A expressed IL-1*β* and IL-6 at levels similar to those detected in PI-ATM, whereas population B expressed IL-6 at 48 fold and CCL2 at 20-fold higher levels relative to PI-ATM (Figure 4). Clearly, populations A and B warrant further characterization to determine their role in the described obesity model.

### 3.5. Rosiglitazone Attenuated the Inflammatory Mediator Profile of the Epididymal Fat

**Figure 5:**
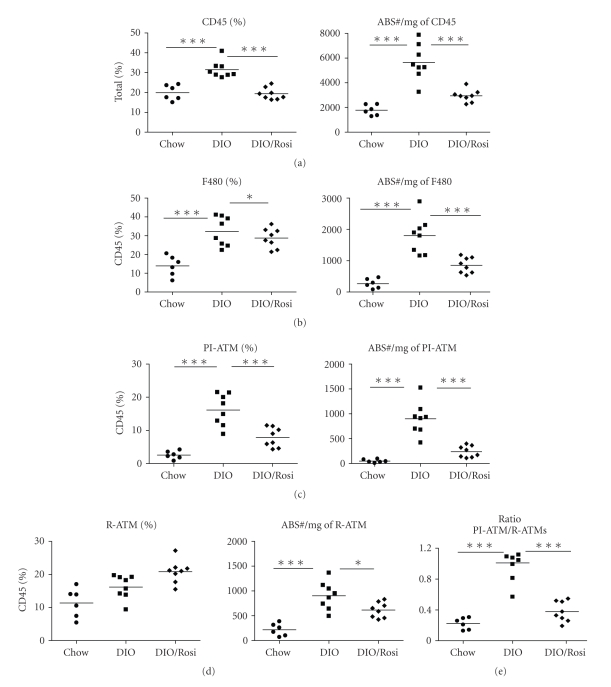
Rosiglitazone reduced the pro-inflammatory ATM infiltration in epididymal fat. Animals were put on Chow or an HF/CH diet for 12 weeks. Then Rosiglitazone at 5 mg/kg or vehicle was supplemented in the food for drug treatment the final 6-weeks. (a)–(d). Flow cytometry was performed at the termination of the study at 18 weeks (*n* ≥ 6 mice/group). (a) Total leukocyte infiltration was assessed by CD45. (b) Total macrophage infiltration was determined by CD45+F480+CD11b+ cells. Finally total macrophages were subdivided into two groups the (c) pro-inflammatory CD45+F480+CD11b+CD11c+ ATMs and (d) resident CD45+F480+CD11b+CD11c ATMs. (e) The ratio of the pro-inflammatory ATMs to resident ATM populations was reduced with Rosiglitazone. Values represent the mean and SEM. The groups are represented by the following symbols: circles (Chow), squares (DIO), and diamonds (DIO/Rosi). One way ANOVA (Bonferroni multiple comparison) was used to compare Chow to DIO and DIO to DIO/Rosi at each time point; *P < .05 and ***P < .001.

**Figure 6:**
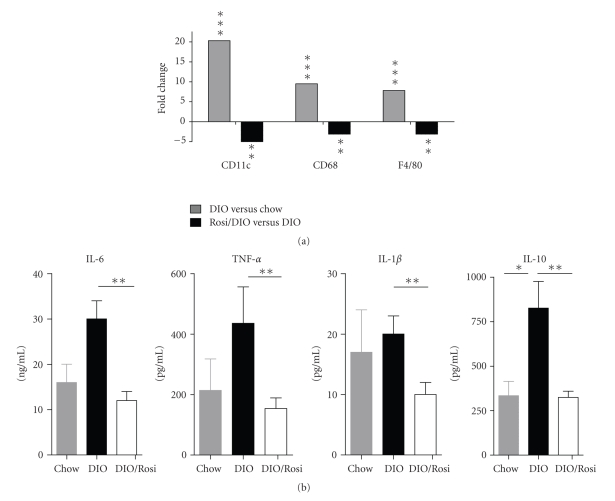
Rosiglitazone reduced macrophage and pro-inflammatory markers by gene expression as well as ex vivo spontaneous mediator production. (a) Gene expression of macrophage markers was analyzed from the epididymal fat pads. Data is represented as DIO gene expression fold induction versus the chow cohort or DIO animals treated with Rosiglitazone versus the DIO cohort (*n* = 8 mice/group). Statistical significance was determined by a two-tailed Welch *t*-test; *P < .05, **P < .01, and ***P < .001. (b) Stromal vascular cells were isolated from the EF and incubated ex vivo. Spontaneous mediator production was assessed (*n* = 8 mice/group). Values represent the mean and SEM. Mann-Whitney *U* test was used to compare standard chow to DIO or DIO to DIO/rosiglitazone; *P < .05 and *P < .01.

Rosiglitazone treatment reduced hyperinsulinemia (DIO 4510 +/− 490 pg/ml: DIO-Rosi 2170 +/− 620 pg/ml), serum CCL2 levels (DIO 84.2 +/− 0.18 pg/ml: DIO-Rosi 47.3 +/− 4.6 pg/ml) and SVF mediator production of IL-6, TNF*α*, IL-1*β* and IL-10 (Figure 6(b)). Rosiglitazone treatment significantly decreased the percentage and absolute number of total leukocytes (Figure 5(a)) and total F480+ macrophages (Figure 5(b)) in the fat tissue. Of the macrophages, rosiglitazone preferentially decreased the proportion of PI-ATM (Figure 5(c)) and elevated the proportion of R-ATM (Figure 5D), which ultimately decreased the PI/R-ATM ratio (Figure 5(e)). Similarly, decreased macrophage accumulation in the rosiglitazone cohort was supported by decreased mRNA levels of F480, CD68 and CD11c in the epididymal fat (Figure 6(a)). The newly identified populations A and B were not followed in the current rosiglitazone study but will be pursued pending further cell characterization.

### 3.6. Rosiglitazone Modulates the HF/CH Diet-Induced Blood Monocytes

**Figure 7:**
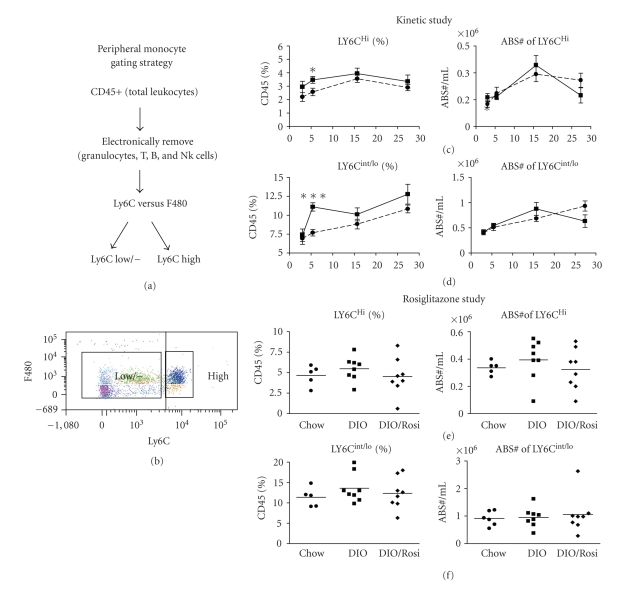
Rosiglitazone can modulate a trend for an elevation in the percentage of monocytes. Whole blood was stained and monocytes were assessed in the kinetic timecourse at 3, 6, 16, and 27-weeks, as well as in the Rosiglitazone study terminated at 18 weeks. (a) and (b) show the gating strategy and a representative dot plot that were employed to identify the monocyte populations. (c)-(d). In the kinetic study, results show a trend for an increase in the percentage of both Ly6Chi and Ly6Clo monocyte populations in the DIO cohort compared to chow, but not in absolute numbers. (e)-(f). Rosiglitazone nonsignificantly reduced the elevation in both the Ly6C high and Ly6Clo monocyte percentages and the Ly6Chi but not Ly6Clo absolute numbers.

Blood monocytes were monitored as a potential biomarker [6, 27, 31, 32]. Similar to the gating strategy for the epididymal fat, CD45 was used to delineate the leukocyte population. Subsequently, Ly6G, CD3, B220, NK1.1, and Thy1.2 were used to identify and electronically discard the neutrophils and T, B, and NK cells (Figures 7(a) and 7(b)). Monocytes were then identified by F480 and Ly6C. Previous reports have classified Ly6C^Hi^ as pro-inflammatory and Ly6C^lo/int   ^ as anti-inflammatory monocytes [32]. Throughout the kinetic study, there is an upward trend in the proportion of both blood monocyte populations in the DIO cohort compared to chow, although the increase only reached significance at 6-weeks (Figures 7(c) and 7(d)). Assessment of the absolute numbers of blood monocytes revealed no kinetic differences observed between the groups (Figures 7(c) and 7(d)). Despite the very modest alteration in the proportion of circulating monocytes in mice on an HF/CH diet, rosiglitazone attenuated the increase in the proportion of Ly6C^Hi^ and Ly6C^lo/int ^ monocytes (Figures 7(e) and 7(f)).

## 4. Discussion

The goal of this study was to assess the effect of an HF/CH diet on the cellular and molecular parameters in DIO mice and the effect of an insulin sensitizing agent, rosiglitazone, on these parameters. Changes in metabolic parameters occurred as early as 6-weeks in DIO mice as evidenced by hyperinsulinemia, increased leptin levels and modestly increased levels of pro-inflammatory mediators, including CCL2 and IL-6, in the serum and in the EF pad. Despite these early changes, dramatic metabolic changes only occurred at and beyond 16-weeks.

Cell infiltration is one of the hallmarks of inflammation; we observed an increase in total leukocytes within the SVF by 3 weeks in DIO mice. Within the leukocyte population, the percentage of total macrophages significantly increased by 6-weeks which was corroborated by F480 and CD68 gene expression data and F480 immunostaining of EF tissue. However, the peak of macrophage accumulation occurred at 16 to 27-weeks correlating with maximal insulin levels. In the DIO mice, although there was a modest increase in the proportion of both PI-ATM and R-ATMs at 6-weeks, there was a dramatic increase in the proportion and absolute numbers of PI-ATM at 16 to 27-weeks. This correlated with significant increase in the PI/R-ATM ratio indicative of a pro-inflammatory microenvironment. Thus, as hypothesized, the HF/CH diet led to an early inflammatory response with kinetics paralleling the peak of the insulin levels and liver damage (ALT). 

Analysis of the EF tissue or spontaneous mediator production from cultured SVF cells derived from DIO mice after 16-weeks on the HF/CH diet revealed an induction of the various pro-inflammatory mediators such as CCL2, TNF*α*, IL-6 or IL-1*β* and the pleiotropic mediator, IL-10. To determine if the macrophages were the primary contributors to this mediator production from the SVF, we sorted four populations: PI-ATM, R-ATM and two additional populations and evaluated their mediator profile by gene expression. The two additional populations were CD45+ F480−CD11b+ CD11c+ cells (A) and CD45−F480−CD11b−Ly6C+/−Thy1.2+ cells (B), which express surface antigens potentially associated with other myeloid or hematopoietic cells. 

Our data indicates that both macrophages and novel cell populations within the SVF fraction contribute to the inflammatory state of the adipose tissue in DIO mice. Gene expression profiling of sorted macrophage subsets confirmed that the PI-ATMs had elevated message levels of the pro-inflammatory mediators, including TNF*α*, IL-6, and IL-1*β*, as well as TGF*β*, a finding consistent with human ATM data [15, 16]. In addition, the PI-ATM also expressed elevated levels of IL-10 and ApoE, which are often perceived as anti-inflammatory mediators. However, both IL-10 and ApoE are pleiotropic molecules and they could be exerting pro-inflammatory effects. For example, overexpression of IL-10 in islet cells in a nonobese diabetic mouse model exacerbates diabetes [33] and, recent data indicate that ApoE can stimulate dendritic cells to present lipids [34]. These mediators could be having pro-inflammatory roles or they could be coexpressed as a mechanism to counter-regulate the pro-inflammatory state. Similar to our data some report the mediator profile of PI-ATMs from mice on HF or Chow diets showed expression of TNF*α*, IL-6 and/or IL-10 [14] or a subset of these mediators [12, 13, 31]. Overall, from the sorted SVF populations, the PI-ATMs described in this manuscript are highly metabolically active, expressing multiple pro-inflammatory mediators (IL-1*β*, IL-6, TNF*α*), growth factors (TGF*β*) and pleiotropic mediators (APOE and IL-10). Although the described ATMs could not be definitively classified as pro or anti-inflammatory, these data are consistent with the human reports indicating that human ATMs [10, 11, 16] and cultured human SVF cells showed elevated levels of IL-1*β*, TNF*α*, IL-6 and CCL2 as well as IL-10 [11, 15]. With the exception of LYVE-1, a gene recently associated with tissue angiogenesis [35] or remodeling [16], the R-ATM described herein expressed lower levels of most mediators relative to the PI-ATM, despite reports of elevated levels of IL-10 [13] and ApoE [12, 31] in these cells. 

Some of the discrepancies between the ATM subsets described herein and those of previous studies could be due to differences in the diets, activation or differentiation state of the sorted populations, or due to the plasticity of macrophage populations [36, 37]. Alternatively, differences in the stringency of the gating method and sort strategies could account for the discrepancies. Our data clearly indicated that there are additional populations of cells that can be identified in the adipose tissue that could contribute to mediator production. 

As stated previously, using our gating strategy, two additional inflammatory cell populations were identified. Population B (CD45−Thy1.2+) was a prominent population representing 5–10% of the total SVF. It was observed that this population expressed extremely high levels of IL-6 and CCL2 that far exceeded those of the PI-ATMs. Based on this initial characterization, these cells could represent any of several cell types including newly recruited monocytes, cells of endothelial origin which can secrete CCL2 and IL-6 [15, 16], stromal cells as seen in human adipose tissue [30, 38–41], early hematopoietic derived cells, or preadipocytes. [30, 42]. Population A (CD45+F480−CD11c+) represented 10–20% of the CD45 cells of the SVF and expressed levels of IL-6 and IL-1*β* similar to those of PI-ATMs. These cells, especially population B, contribute to the pro-inflammatory state of the fat tissue. Clearly, as indicated by the profiling of populations A and B, the PI-ATM and R-ATM are not the only cells producing inflammatory mediators in the SVF. Future studies are being designed to further the characterization and to determine if anti-inflammatory or obesity-related drugs alter these populations.

The insulin sensitizing agent, rosiglitazone, is a PPAR*γ* agonist used in the clinic to treat type II diabetes and recently has been reported to affect the phenotypic switch of macrophages from a pro to an anti-inflammatory profile as well as to affect macrophage infiltration into the EF pad [27]. Consistent with reports from clinical studies [24], treatment of DIO mice on the HF/CH diet with rosiglitazone increased the body weight and decreased hyperinsulinemia demonstrating the drug's insulin sensitizing effects (data not shown). These changes in metabolic parameters were paralleled by changes in the pro-inflammatory state of the mice. There was a reduction in spontaneous mediator production from cultured SVF cells from rosiglitazone treated DIO mice which correlated with a decrease in macrophage accumulation. 

 Gene expression analysis indicated a reduction in the macrophage markers, F480 and CD68, as well as CD11c in total adipose tissue from rosiglitazone treated DIO mice. Flow cytometry analysis corroborated and extended these findings by revealing that rosiglitazone treatment significantly reduced the PI-ATMs, lowering the PI/R-ATM ratio to a level close to that observed for the normal Chow cohort. Our data supports the theory that rosiglitazone switches the profile of the ATMs from a pro-inflammatory to a more anti-inflammatory or resting state [43]. Alternatively, rosiglitazone could alter the balance of cell recruitment and migratory clearance of ATMs; in an atherosclerosis model reduced emigration of myeloid derived cells out of plaques contributed to the disease pathology [44].

In addition to the analysis of adipose tissue ATM, peripheral blood monocytes were analyzed as a potential biomarker for obesity and insulin resistance in preclinical and clinical studies. In the kinetic study, we detected a significant elevation in both the pro-inflammatory (Ly6Chi F480+/−) and homeostatic (Ly6Clo/− F480+/−) monocyte populations at 6-weeks. At later time points, there was a trend only for an increase in the percentages but not in number of monocytes consistent with previous reports indicating modest but significant elevations of monocyte populations [27, 32]. Continual emigration from the bone marrow coupled with the simultaneous and continual influx into the fat depots or sites of inflammation may account for the very modest increase in monocytes throughout the treatment period. Despite the limited modulation in monocytes, at 18 weeks post-HF/CH diet, rosiglitazone induced a modest but nonsignificant reduction in the percentage of both monocyte populations. Although it remains possible that rosiglitazone may have had a more dramatic effect on monocyte populations at an earlier time point, 18 weeks represents the peak of the metabolic changes and PI-ATM infiltration. Due to the modest therapeutic window for modulation of monocytes, we concluded that monitoring of monocyte populations in the blood would be inadequate to follow as a biomarker unless a more dramatic modulation of monocytes is observed in human subjects. 

 In conclusion, an HF/CH-like diet rich in fat and cholesterol induces obesity which correlates with an inflammatory state as exhibited by a rapid increase in epididymal fat pad mass, hyperinsulinemia, macrophage infiltration and mediator production in the fat depots. The increased ratio of PI/R-ATMs and the production of many pro-inflammatory mediators correlated with the peak of disease induced by the HF/CH diet as evidenced by maximal insulin levels at 16-weeks and are indicative of an inflammatory state in DIO mice. At the peak of disease, rosiglitazone attenuates the inflammatory response as exemplified by a reduction in the ratio of PI/R-ATMs and the production of multiple mediators. Lastly, a very intriguing finding was the identification of a novel population in the SVF that expressed very high mRNA levels of IL-6 and CCL2 exceeding those observed in the PI-ATM. This population warrants additional investigation as it could significantly impact the inflammatory state in DIO mice and potentially in human obese and diabetic subjects. This DIO mouse model induced with HF/CH is an appropriate model for characterization of therapeutics targeted not only for the metabolic parameters of the syndrome but also for the low-level chronic inflammatory response that is induced by the diet.

## Supplementary Material

Supplementary Material includes a Supplementary Table and a Supplementary Figure in addition to an illustration of the immunohistochemistry.

## Figures and Tables

**Table 1:** Composition of rodent diets.

	Chow diet	HF/CH diet
Ingredients (% kcal)		
Protein	24.7	20
Carbohydrates	62.1	35
Fat	13.2	45
Total	100	100
kcal/gm	3.85	4.72

Total cholesterol (% of diet)	0	0.12

Chow: Cat # 5053 PicoLab Rodent Diet 20.

HF/CH: high-fat/cholesterol diet; Cat # D04012801 Research Diets Inc.

**Table 2:** Elevated serum metabolic parameters and inflammatory mediators in the DIO cohort.

	6-weeks		16-weeks		27-weeks	
Parameter	CHOW	DIO	CHOW	DIO	CHOW	DIO
Insulin, pg/ml	57.8 (41)	383.4 (76)*	710.9 (145)	2901.0 (716)*	1458 (446)	2602.5 (289)*
ALT, U/ml	23.0 (1.1)	29.5 (2.0)*	29.1 (5.3)	174.2 (40.9)*	22.9 (1.9)	82.7 (11.6)*
Leptin, ng/ml	0.69 (0.30)	58.8 (14.6)*	7.1 (1.5)	32.9 (12.5)*	4.6 (1.2)	57.0 (12.6)*
CCL2, pg/ml	0	4.3 (1.1)	18.9 (2.4)	42.1 (3.4)*	168.6 (21)	234.7 (14.4)*
IL-6, pg/ml	0	3.5 (0.6)	9.6 (0.7)	25.5 (7.7)^#^	32.7 (11.2)	54.1 (17)
TNF, pg/ml	15.9 (2.1)	19.9 (2.3)	13.2 (0.4)	13.2 (0.5)	1.6 (0.7)	1.5 (0.3)
IL-1b, pg/ml	15.9 (2.1)	19.9 (2.3)	34.8 (1.4)	37.4 (1.5)	0.9 (0.2)	1.1 (0.2)
IL-10, pg/ml	21.8 (9.2)	33.1 (6.8)	39.1 (1.8)	66.8 (16.4)	22.1 (7.3)	69.8 (33)

At each timepoint, mice from separate groups of animals were terminally bled (Chow *n* = 8–13 mice and the DIO *n* = 7–18 mice). Values represent the mean and SEM. Mann-Whitney *U* test was used to compare Chow to DIO at each time point; *P < .05 and ^#^ *P* < .08.

## Abbreviations

DIO: diet induced obesity
HF/CH: high-fat (45%) and cholesterol (0.12%)
BMI: body mass index
IR: insulin resistance
BW: body weight
PPAR*γ*: peroxisome proliferator-activated *receptor* gamma
EF: epididymal fat
SVF: stromal vascular fraction
ATM: adipose tissue macrophage
R-ATM: resident adipose tissue macrophage
PI-ATM: pro-inflammatory adipose tissue macrophage
TZD: thiazolidinedione drugs
ALT: alanine aminotransferase
NAFLD: nonalcoholic fatty liver disease
ITT: insulin tolerance test
GTT: glucose tolerance test.
