# Supplementary material for: Kinetic Assessment and Therapeutic Modulation of Metabolic and Inflammatory Profiles in Mice on a High-Fat and Cholesterol Diet
Source: PPAR Res. 2010 Apr 22;2010:970164. doi: 10.1155/2010/970164 (PMC2859407; doi:10.1155/2010/970164)
Supplement: Supplementary file 1 — Supplementary Material includes a Supplementary Table and a Supplementary Figure in addition to an illustration of the immunohistochemistry. [file 970164.f1.pdf]

Supplemantal Table 1. Primer sequences

Gene	Refseq ID	Forward Primer	Reverse Primer	Probe (UPL)	Probe	Control (PCR fragment)
IL-1β	NM_008361	142/ttgacggaccccaaaagat	216/gaagctggatgctctcatctg	# 26	ctgggctg	98-598 (501 bp)
IL-6	NM_031168	327/gctaccaaactggatataatcagga	404/ccaggtagctatggtactccagaa	# 6	ttcctctg	65-542 (478 bp)
TNFα	NM_013693	428/ctgtagcccacgtcgtagc	524/ttgagatccatgccgttg	# 25	tggaggag	281-699 (419 bp)
IL-10	NM_010548	184/cagagccacatgctcctaga	262/tgtccagctggtcctttgtt	# 41	cttcagcc	92-497 (406 bp)
CCL2	NM_011333	142/catccacgtgttggctca	217/gatcatcttgctggtgaatgagt	# 62	acctgctg	36-451 (416 bp)
TGFβ	NM_011577	1358/tggagcaacatgtggaactc	1428/cagcagccggttaccaag	# 72	ttcctggc	1054-1599 (546 bp)
CD11c	NM_021334	1725/atggagcctcaagacaggac	1787/ggatctgggatgctgaaatc	# 20	ccagccag	1554-2145 (592 bp)
LYVE-1	NM_053247	758/gaagcagctgggtttgga	847/gtagcaaacagccagcacag	# 91	ctctcctc	623-1092 (470 bp)
ApoE.	NM_009696	370/cccaagtcacacaagaactgac	465/ccactggacccagctgtt	# 17	aggagctg	186-617 (432 bp)
