# Supplementary material for: Kinetic Assessment and Therapeutic Modulation of Metabolic and Inflammatory Profiles in Mice on a High-Fat and Cholesterol Diet
Source: PPAR Res. 2010 Apr 22;2010:970164. doi: 10.1155/2010/970164 (PMC2859407; doi:10.1155/2010/970164)
Supplement: Supplementary file 2 [file 970164.f2.pdf]

Supplemental Materials and Methods

Immunohistochemistry (IHC)

Adipose tissue samples were fixed for 20 hours at room temperature in 10% formalin fixative, processed and embedded in paraffin. Five-micrometer sections were collected, deparaffinized in xylene, rehydrated and treated with trypsin for antigen retrieval. The sections were stained for expression of F4/80 with an anti-F4/80 (CI:A3-1) monoclonal antibody (Serotec Raleigh NC). Visualization of F4/80 staining was carried out with an alkaline phosphatase system (Vector Burlingame, CA) using liquid permanent red as the substrate (Dako; Carpinteria, CA). Hematoxylin (Dako) was used to counterstain the sections.

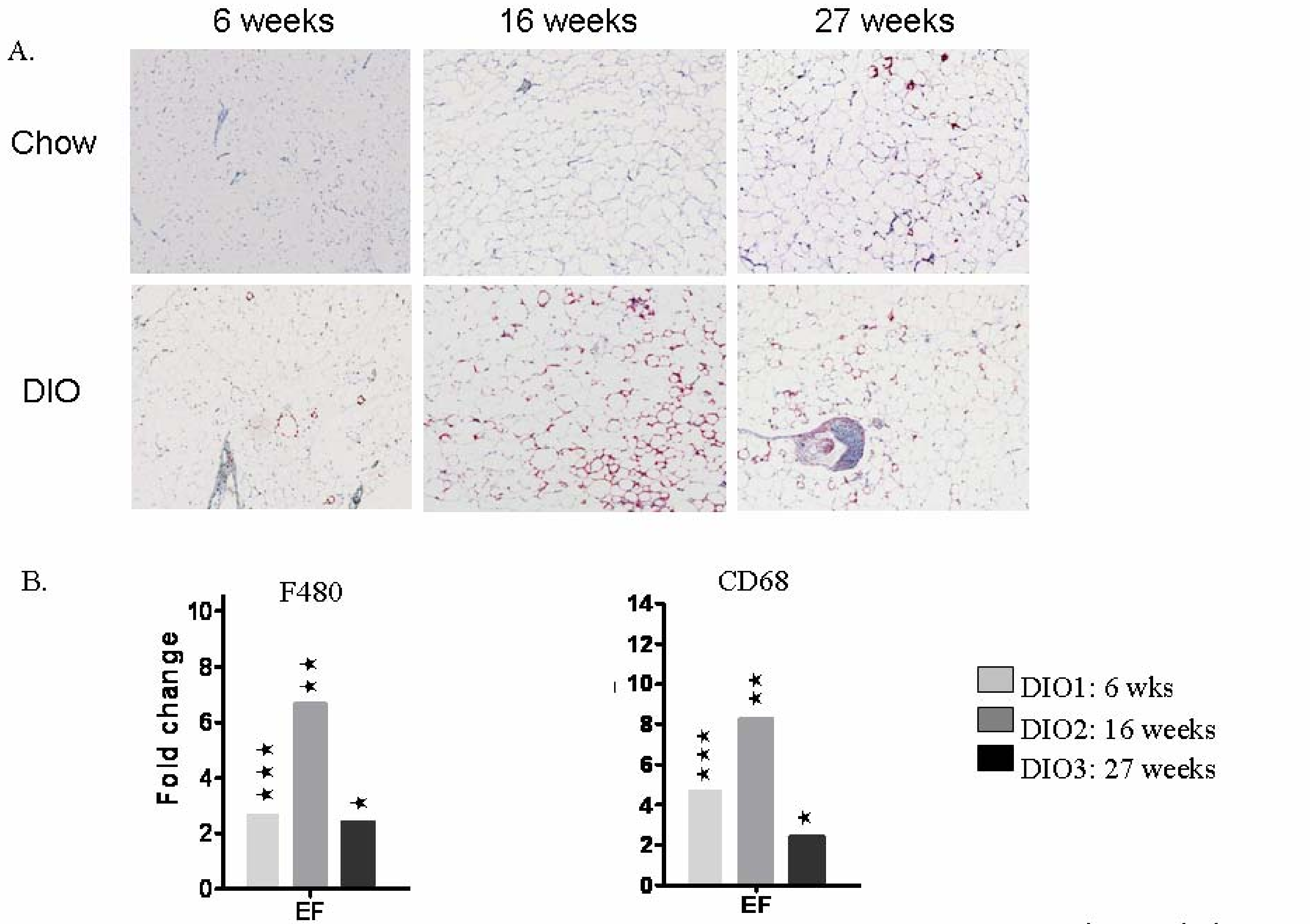

Supplemental Figure 1
